# Cdc6 cooperates with c-Myc to promote genome instability and epithelial to mesenchymal transition (EMT) in zebrafish

**DOI:** 10.18632/oncotarget.2204

**Published:** 2014-07-11

**Authors:** Ching-Hung Chen, Dar-Shong Lin, Chieh-Wen Cheng, Chun-Ju Lin, Yu-Kang Lo, Chueh-Chuan Yen, Alan Yueh-Luen Lee, Chung-Der Hsiao

**Affiliations:** ^1^ Department of Bioscience Technology, Chung Yuan Christian University, Chung-Li, Taiwan; ^2^ Department of Pediatrics, Mackay Memorial Hospital, Taipei, Taiwan; ^3^ Department of Medical Research, Mackay Memorial Hospital, Taipei, Taiwan; ^4^ Mackay Junior College of Medicine, Nursing, and Management, Taipei, Taiwan; ^5^ Mackay Medical College, Taipei, Taiwan; ^6^ National Institute of Cancer Research, National Health Research Institutes, Miaoli, Taiwan; ^7^ Division of Hematology & Oncology, Department of Medicine, Taipei Veterans General Hospital, Taipei, Taiwan; ^8^ National Yang-Ming University School of Medicine, Taipei, Taiwan; ^9^ Therapeutical and Research Center of Musculoskeletal Tumor, Taipei Veterans General Hospital, Taipei, Taiwan; ^10^ Center for Nanotechnology, Chung Yuan Christian University, Chung-Li, Taiwan

**Keywords:** Cdc6, c-Myc, zebrafish, genomic instability, epithelial to mesenchymal transition

## Abstract

Aberration in DNA replication is a major cause to genome instability that is a hallmark of cancer cells. Cell division cycle 6 (Cdc6) and c-Myc have a critical role in the initiation of DNA replication. However, whether their interaction induces epithelial-mesenchymal transition (EMT) and promotes tumorigenesis in *in vivo* animal model remains unclear. Since using zebrafish as a cancer model has been restricted by the late onset of tumorigenesis and extreme difficulty in transformation on skin, we tried to establish a novel non-melanoma skin model in zebrafish to study their role in tumorigenesis. A stable transgenic zebrafish was created by using tol2 transposon, in which cdc6 and c-myc were co-overexpressed in epidermis driven by a skin-specific *krt4* promoter. Intriguingly, co-overexpression of cdc6 and c-myc in transgenic zebrafish skin triggered tumor-like transformation, apoptosis attenuation, genomic instability, and EMT, hallmarks of malignant tumorigenesis. Our findings and other characteristics of zebrafish, including optical clarity and small molecule treatment, provide the future utility of this model for easy and non-invasive detection and for identification of new anti-cancer drug.

## INTRODUCTION

DNA replication is initiated by coordinated binding and highly controlled activation of the prereplicative complex (pre-RC). The pre-RC is called a replication licensing machinery including two replication licensing factors, Cdt1 and Cdc6 that are recruited during G1-S transition on the origin recognition complexes and promote the loading of mini-chromosome maintenance (MCM) complex on chromatin [1, 2]. Deregulation of normal replication mechanism can create a cellular environment, termed replication stress [3], prone to genomic instability that is a hallmark of cancer [4]. Indeed, high levels of Cdc6 and Cdt1 have been observed in various types of cancer [5-8]. Cdc6 has been reported to interact with c-Myc that increases the replication activity with subsequent DNA damage [9]. However, the ability of Cdt1 and Cdc6 overexpression leads to rereplication, providing seeds for chromosome instability, has been recently challenged [10]. In addition, overexpression of Cdc6 induced by Ras or Mos oncogenes causes replication stress that activates DNA damage response (DDR)-mediated senescence [11, 12]. In addition to the licensing factor mechanism, Cdc6 interacts with c-Myc to repress E-box-dependent transcription by interfering with the formation of c-Myc/Max complex [13]; it exert its oncogenic activity by repressing the *INK4/ARF* [7] and *CDH1* locus [14] that encodes E-cadherin, a marker of epithelial to mesenchymal transition (EMT). However, the issues of whether their interaction induces EMT and whether the interaction promotes cancer development in *in vivo* animal model remain obscure.

The zebrafish has become a good lower vertebrate model for studying carcinogenesis in recent years. Compared to other higher vertebrate models, zebrafish model advantages its rapid development, fecundity, amenability to both forward and reverse genetic strategies, body transparency, and suitability to *in vivo* imaging and chemical screening. Importantly, tumor in zebrafish resembles various types of human tumors at either the histological level [15] or cancer characteristics such as genomic instability, neoplastic cell transformation, and oncogene activation [16, 17]. For instance, several loss-of-function of tumor suppressors, such as tp53 [18], PTEN [19] and APC [20], or gain-of-function of oncogenes, c-myc [21], Akt2 [22], and K-ras [23], have been established in zebrafish, which showed similar phenotypes to human cancer.

Although great advances have been achieved in zebrafish cancer model, several obstacles still need to be overcome in order to develop zebrafish as a promising and complementary cancer model. First, the onset of tumor transformation is quite late and usually occurs during juvenile to adult stages. For example, the first appearance of liver cancer in zebrafish is around 3 to 4 weeks post-fertilization after deregulated activation of oncogenes [24]. Second, some tissues like skin proceed oncogenic transformation with extreme difficulty. Third, since the body transparency at adult stage is reduced, it is hard to detect tumor formation and metastasis at adult stage by appearance. Therefore, how to induce the early onset of cancer formation on skin and develop an easy, non-invasive detection methodology are two key issues to the establishment of zebrafish cancer model.

In this study, we aimed to create a novel non-melanoma skin cancer model in zebrafish by co-overexpression of Cdc6 and c-Myc to study their oncogenic mechanisms through the observation on an early onset and visible transformation on the outmost skin layer. To generate the transgenic zebrafish model, we used tol2 transposon tool to overexpress cdc6 and c-myc in epidermis driven by a skin-specific *krt4* promoter. Here we successfully created the stable zebrafish transgenics carrying cdc6 and c-myc. Furthermore, we investigated the underlying mechanism of cellular transformation triggered by co-overexpression of Cdc6 and c-Myc using a transgenic zebrafish skin model, which is involved in accelerated cell proliferation, apoptosis attenuation, genomic instability, and EMT.

## RESULTS

### Single overexpression of cdc6 or c-myc is not sufficient to oncogenic transformation of zebrafish skin

To address the role of interaction between Cdc6 and c-Myc in tumorigenesis *in vivo*, we chose zebrafish as a model to study multiple-oncogenic transformation on the zebrafish skin. To determine the gain-of-function of Cdc6 and/or c-Myc on epidermal transformation in zebrafish, we constitutively overexpressed human cdc6 (hcdc6) and mouse c-myc T58A (m.c-myc T58A) genes in epidermis under the control of keratin4 (*krt4*) promoter (Fig. S1A). The *cmlc2-EGFP*-pA mini-cassette encodes a cytoplasmic-targeted EGFP, being a reporter for identification of transgenics. We successfully isolated four lines carrying cdc6 (germ-line transmission rate = 7%) and 28 lines carrying c-myc (61%). Among all lines, Tg(krt4:hcdc6)^cy24^ and Tg(krt4: m.c-myc T58A)^cy21^ displayed the most strongest green fluorescence in the heart and were kept for the following experiments. We used genotyping to identify stable krt4-cdc6 or c-myc expression according to positive genomic PCR results in the F1 transgenic progeny (Fig. S1A).

**Figure 1:**
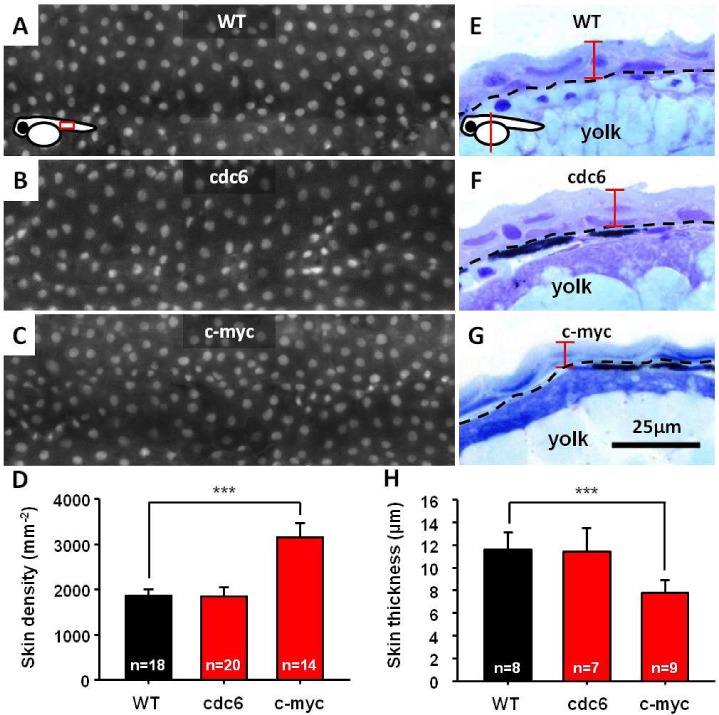
Overexpression of either cdc6 or c-myc is insufficient to transform zebrafish skin cells (A-D) Comparison of the skin cell density between WT, cdc6- and c-myc-overexpressing fish embryos aged 3 dpf. To facilitate skin cell counting, WT, cdc6- and c-myc-overexpressing fish are cross with Tg(*krt4*:nlsEGFP)^cy34^ and the skin cell density was compared for the embryos aged 3 dpf. (E-H) Comparison of the skin cell thickness between WT, cdc6- and c-myc-overexpressing fish embryos aged 3 dpf. WT, cdc6- and c-myc fish embryos were embedded in resin and conducted semi-thin section at 3 μm intervals. The length of skin thickness calculated is highlighted by red color. Scale bar in = 25 μm. The significant difference of skin cell density (D) and thickness (H) between embryos were conducted with t-test.

However, we were unable to observe any oncogenic transformation on skin when either cdc6 or c-myc transgene was overexpressed. To clarify this observation, we sought to compare the density of skin cell between transgenics and WT. To quantitate skin density, Tg(krt4:hcdc6)^cy24^ and Tg(krt4: m.c-myc T58A)^cy21^ were crossed with Tg(krt4:nlsEGFP)^cy34^, and the number of EGFP-positive skin cell nucleus in the trunk region was counted. The EGFP-positive cells enable us to calculate the skin cell number and density in a single cell resolution. We evaluated the skin cell density and morphology on embryos aged 3 day post-fertilization (dpf). Results showed that when cdc6 was overexpressed, there is no significant difference of skin cell density between WT (1868±154 mm^−2^) and Tg(krt4: krt4:hcdc6)^cy24^ (1850±211 mm^−2^) (Figs. 1A, B, and D). However, c-myc overexpression significantly increased the skin density (3169±312 mm^−2^) compared with WT (1868±154 mm^−2^) (Figs. 1A, C, and D). We next observed the skin morphology at histological level by using plastic section through the yolk sac region. We found that the skin of Tg(krt4: m.c-myc T58A)^cy21^ (7.7±1.2 μm) is significantly thinner than WT (11.6±1.5 μm) and Tg(krt4: krt4:hcdc6)^cy24^ (11.4±2.1 μm) (Figs. 1E to G and H). These results clearly suggested that neither Cdc6 nor c-Myc overexpression only is sufficient to transform zebrafish skin although c-Myc induces skin hyperplasia.

### Co-overexpression of cdc6 and c-myc induces tumor-like transformation in zebrafish skin

**Figure 2:**
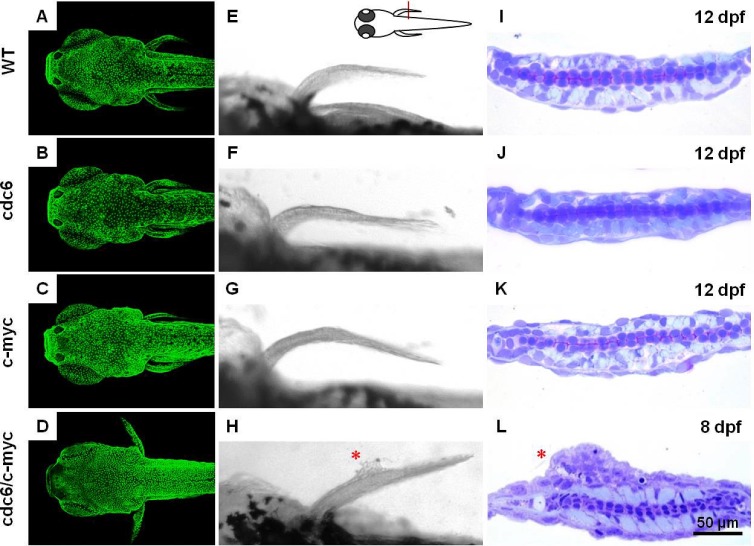
Co-overexpression of cdc6 and c-myc successfully transforms zebrafish skin cells (A-D) Comparison of the skin cell density between different genetic backgrounds which carrying either (A) WT, (B) cdc6, (C) c-myc or (D)cdc6/c-myc transgenes. (E-H) Comparison of the gross morphology in the pectoral fin area in either (E) WT, (F) cdc6, (G) c-myc or (H) cdc6/c-myc transgenes. (I-L) Comparison of the histology in the pectoral fin area in either (I) WT, (J) cdc6, (K) c-myc or (L) cdc6/c-myc transgenes. The developmental stage that the embryos were sacrificed for histology assessment was labeled in the upper right corner. Embryos were crossed sectioned across the pectoral fins positions (highlighted by red line) and the sections were counterstained with H&E. Scale bar = 100 μm in the bright field images (left panel in all images). Scale bar = 50 μm in the plastic sections (right panel in all images). In images from A to H, anterior is positioned to the left.

The failure to transform zebrafish skin in Tg(krt4:hcdc6)^cy24^ and Tg(krt4: m.c-mycT58A)^cy21^ leads us to test whether co-overexpression of cdc6 and c-myc transforms zebrafish skin. The skin out-looking of progenies derived from either single or double transgenic line with nlsEGFP background was monitored by using the fish aged from 3 to 12 dpf. After examining skin morphology and histology, we were unable to detect any sign of skin oncogenic transformation in WT (Figs. 2A, E, I), cdc6 (Figs. 2B, F, J), or c-myc single transgenic lines at 12 dpf (Figs. 2C, G, K). However, about 15% progenies derived from cdc6 and c-myc double transgenic lines displayed tumor-like protruding structure on the skin surface (especially on the pectoral fin) from at only 8 dpf (Figs. 2D, H, L, S1B). In addition, all progenies with skin tumor phenotype were lethal and not survived longer than 15 dpf. Why is pectoral fin region so sensitive to skin tumor-like transformation? We hypothesized that the cell proliferative kinetics in the pectoral fin is much faster than the one in other tissue compartments. To prove this hypothesis, we performed BrdU incorporation (S phase marker) and pHistone3 staining (M phase marker) on cdc6/c-myc transgenics aged 8 dpf. Results support our speculation since the robust BrdU- or pH3-positive cells were observed in largely the domain of pectoral fin (Fig. S2A-D). In addition to the pectoral fin, we found that the anal and tail fins are a hot spot for active skin cell proliferation (Fig. S2E).

### Co-overexpression of cdc6 and c-myc induces skin hyperplasia by increasing cell proliferation and attenuating apoptosis

**Figure 3:**
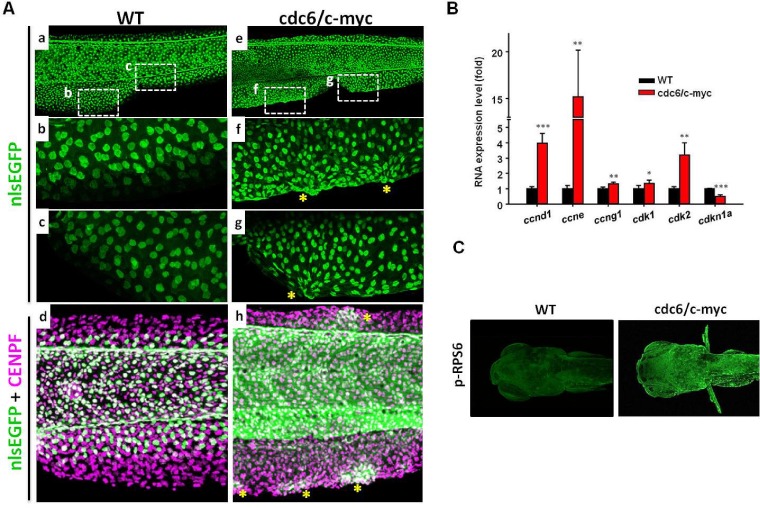
The skin cell morphology and cell proliferation activity in cdc6/c-myc transgenic fish (A). The skin cell morphology in the anal and tail fin regions of WT (a-d) or cdc6/c-myc double transgenics (e-h) aged 8 dpf. To facilitate skin cell counting, WT, cdc6- and c-myc-overexpressing fish were crossed with Tg(*krt4*:nlsEGFP)^cy34^ and their skin cell density were compared. The positions labeled by dotted line in the anal (f) and tail (g) fin of cdc6/c-myc transgenics were enlarged to show abnormal skin aggregation in cdc6/c-myc transgenics. The corresponding positions in WT were also shown (b and c). Total cell nuclei including skin cells were labeled by CENPF antibody (magenta color) (d and h) and skin cells were double labeled by CENPF and EGFP antibody (green color). Overexpression of cdc6/c-myc in skin cell can induce skin cell abnormal aggregation in dorsal and anal fin (stars in f, g, and h). (B) Comparison of the expression of cell cycle markers by real time RT-PCR. The cell cycle markers like ccnd1, ccne, and cdk2 were significantly upregulated while the cell cycle inhibitor cdkn1a was significantly downregulated. (C) Comparison of the protein expression of protein synthesis markers. The marker like p-RPS6 was greatly upregulated in cdc6/c-myc transgenics. Result is presented as a representative of at least three independent experiments.

**Figure 4:**
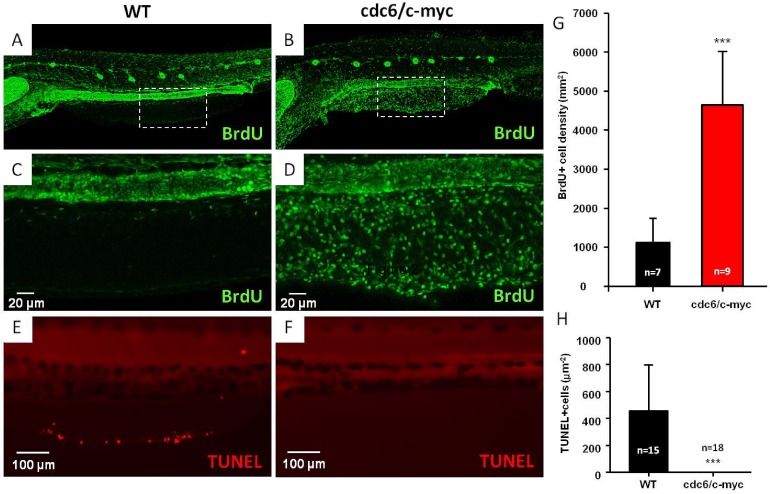
Comparison of cell proliferation and apoptosis in WT and cdc6/c-myc transgenic zebrafish The BrdU incorporation experiment was conducted to compare the cell proliferation index between WT (A and C) and cdc6/c-myc double transgenic fish (B and D). Embryos aged 7 dpf were incubated with 10 mM BrdU for 24 hr and later washed with fish water and finally fixed with PFA at 8 dpf and proceed for anti-BrdU antibody staining. The images highlighted by dotted lines in the ventral fin (A and B) were magnified in C and D, which show the cell patterns with positive BrdU-labeling. The area of BrdU + cells was quantified by image J software and statistically presented in (G) for comparison. Scale bar =20 μm in C and D. The TUNEL assay was conducted to compare the apoptotic index between WT (E) and cdc6/c-myc double transgenic fish (F). The area of TUNEL+ cells was quantified by image J software and statistically presented in (H) for comparison. Scale bar =100 μm in E and F. In all images, anterior is positioned to the left. The significance was compared with student T-test.

The intriguing oncogenic transformation phenotype in the skin of cdc6/c-myc double transgenics leads us to ask whether this is caused by acceleration of cell cycle and attenuation of cell death. High magnification view of the anal and tail fins showed that skin cells are aggregated in the fin fold regions of cdc6/c-myc transgenics (e to h in Fig. 3A), while those in the WT fish are deposited in an organized order (a to d in Fig. 3A). By using anti-GFP (krt4-positive cells) and anti-CENPF (cell nucleus labeling) double staining, krt4-positive cells were observed in the entire region of fin fold in cdc6/c-myc transgenic embryos (h in Fig. 3A), which might be caused by the migration of the cells from lateral epidermis to fin folds. However, in the wild-type embryos, we found that krt4-positive cells were only partially distributed in the fin fold region but never observed in the marginal region of the same area (d in Fig. 3A). To confirm active cell proliferation in skin cells from cdc6/c-myc transgenics at molecular level, we examined the gene expression of cell cycle-related markers. Results showed that cyclin D (*ccnd1*), cyclin E (*ccne*), cyclin G1 (*ccng1*), cyclin-dependent kinase 1 (*cdk1*), and CDK 2 (*cdk2*) were upregulated, while CDK inhibitor 1a *(cdkn1a*, p21/WAF1) was downregulated in the cdc6/c-myc transgenics (Fig. 3B). In addition, we noticed that the expression level of p-RPS6, a marker for protein translation, was highly upregulated in cdc6/c-myc lines, suggesting that protein synthesis is highly active in cdc6/c-myc transgenic fish (Fig. 3C). We next further quantitated the skin cell proliferation in either WT or double transgenics aged 8dpf by BrdU incorporation using an image-based cytometry. Results showed that the cell proliferation activity in cdc6/c-myc transgenics was much higher than the one in WT (Fig. 4A, B, G and S3). For example, a high magnification view in the ventral fin region showed that the BrdU-positive cells in cdc6/c-myc double transgenics were higher as 13 folds than the ones in WT (Fig. 4C and D). We concluded that co-overexpression of cdc6 and c-myc in zebrafish skin greatly accelerates cell proliferation.

To address apoptosis attenuation, we performed a whole-mount fluorescent TUNEL staining using 8dpf aged embryos of either WT or double transgenics. Results showed that physiological amounts of apoptotic cells were observed in the anal fin margins of WT (Fig. 4E) but not in cdc6/c-myc transgenics whose apoptotic cells were significantly abolished (Fig. 4F and H). Taken together, we concluded that co-overexpression of cdc6 and c-myc in zebrafish skin accelerates cell proliferation and attenuates apoptotic cell death, finally giving an increase in the hyperplastic growth of skin cells.

### Detection of nuclear aberration and genomic instability in cdc6/c-myc double transgenics

**Figure 5:**
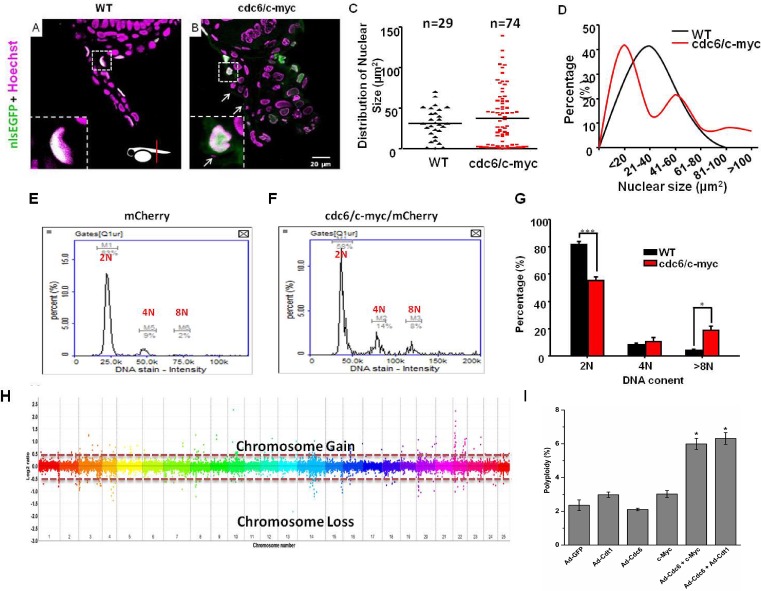
cdc6/c-myc co-overexpression induces polyploidy and chromosomal instability in transgenic zebrafish and human cell Detection of skin morphology in the pectoral fin region of WT (A) and cdc6/c-myc (B) transgenic zebrafish embryos aged at 8 dpf. The skin cells were visualized by nlsEGFP siganls (green color) and the cell nuclei were counterstained by Hoechst staining (pseudo-color in magenta). The normal skin cell nucleus was shown in flat and distributed in the outermost layer (A). The micronuclei and nucleus aberration were observed in the skin of ventral fin region of cdc6/c-myc double transgenics (B). The micronuclei (highlighted by arrows) were magnified and highlighted in the left lower corner. (C) The distribution of nuclear size in WT and cdc6/c-myc double transgenics. (D) The percentage of nuclear size in WT and cdc6/c-myc double transgenics. Scale bar = 20 μm in A and B. Comparison of the DNA content in skin cells derived from either WT (E) or cdc6/c-myc double transgenic fish (F) aged at 8 dpf by using image based cytometry (N=10). (G) Statistic analysis of different DNA content in skin cells derived from WT and cdc6/c-myc double transgenic fish. Result showed that the 2N content was decreased by 26.34% and the > 8N content increased by 14.6%. (H) Evaluations of the chromosomal stability of WT and cdc6/c-myc double transgenic fishes aged 8 dpf by using array CGH. (I). Co-overexpression of Cdc6 and c-Myc in human cells induces polyploidy. U2OS cells were infected with Ad-Cdc6 (1.2 × 10^7^ pfu/ml), Ad-Cdt1 (1.2 × 10^7^ pfu/ml) and/or Ad-Vec (Ad-GFP, 1.2 × 10^7^ pfu/ml) and/or transfected with c-Myc expression plasmid. The cell cycle profile was obtained by FACS analysis 48 h after infection or transfection. The percentage of cells containing more than 4 N of DNA content is calculated and presented as polyploidy.

Overexpression of Cdc6 and c-Myc leads to polyploidy and genomic instability in a variety of cancers [5, 6, 9, 25]. To examine the transformation phenotype in zebrafish skin, we performed plastic section through the tumor-like structure in the anal fin area of cdc6/c-myc transgenics. Compared to WT, the nucleus morphology of cdc6/c-myc fish displays severe abnormality (Fig. 5A and B). By measuring the nuclear size of the skin cells, nuclear pleomorphism was detected in cdc6/c-myc transgenics. In WT, the nuclear size displays normal distribution pattern with an average nuclear size at 31±3 μm^2^. However, only in cdc6/c-myc transgenics, some macronucleoli (nuclear size larger than 100 μm^2^) and micronucleus (nuclear size smaller than 10 μm^2^) were detected (Fig. 5C and D).

The nuclear atypia phenotype detected in cdc6/c-myc transgenic embryos strongly suggests it may accompany with genomic instability problem. To validate this hypothesis, we performed image-based cytometry and array-based comparative genomic hybridization (aCGH) to detect the DNA content and chromosome stability of the transformed skin, respectively. For DNA content analysis, cdc6/c-myc transgenic embryo cells with H2AFZ-mCherry background aged 8dpf were gently dissociated, stained with Hoechst 33342 nuclear dye, and then subjected to image-based cytometry. For WT, both the skin and non-skin cell fractions display normal DNA content histogram (Fig. 5E and G). However, in cdc6/c-myc transgenic embryos, skin cells display typical polyploidy that is characterized with abnormal accumulation of 8N fractions (Fig. 5F and G). To further confirm genome instability in the zebrafish skin cells, we isolated genomic DNA from WT and cdc6/c-myc transgenic embryos aged 8 dpf, labeling genomic DNA with Cy3 and Cy5 dyes. The genomic DNA then was subjected to perform aCGH analysis with zebrafish 385K CGH microarray. Results showed that cdc6/c-myc transgenic embryos have abnormal subchromosomal amplification in Chromosome 10 and 22 (Fig. 5H).

To confirm the phenomenon of polyploidy observed in the zebrafish model, we tried to co-overexpress Cdc6 and c-Myc in human cells. After infecting cells with recombinant adenoviruses (Ad-Cdc6 or Ad-Cdt1), rereplication was monitored by accumulation of cells containing more than 4N DNA content as described [26], which presents a form of replication stress, fuelling polyploidy and genomic instability. Co-overexpression of Cdc6 with c-Myc or Cdt1 significantly induced DNA rereplication (Fig. 5I). These results support the idea that co-overexpression of cdc6 and c-myc induces polyploidy and genomic instability, hallmarks of malignant tumorigenesis.

### Detection of ectopic skin cells and EMT in cdc6/c-myc double transgenics

**Figure 6:**
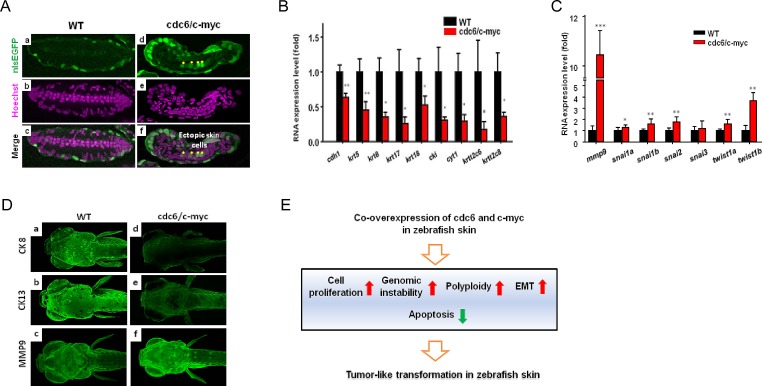
Deregulation of EMT markers and detection of polyploidy in the pectoral fin of cdc6/c-myc transgenic zebrafish and in human cancer cells (A). The skin cells of WT (a-c) and cdc6/c-myc double transgenics (d-f) aged 8 dpf were labeled by nlsEGFP (green color) and co-staining with Hoechst 33342 nuclear dye in the pectoral fin sections. In normal fish, skin cells were restrictedly located in the outmost layer (a, c). However, some ectopic nlsEGFP+ cells (indicted by asterisks) were mixed with the inner cartilage tissues (d, f). (B) Real-time PCR results showed the epidermal markers of keratin gene family were significantly downregulated in cdc6/c-myc double transgenic embryos aged 8 dpf. (C) Real-time PCR results showed the mesenchymal markers were deregulated in cdc6/c-myc double transgenics. MMP9 was highly expressed in cdc6/c-myc double transgenics. (D) Comparison of the EMT marker expression at protein levels. Epidermal markers like CK8 (a, d) and CK13 (b, e) were greatly downregulated while mechenchymal marker like MMP9 (c, f) was highly upregulated in cdc6/c-myc double transgenics. Result is presented as a representative of at least three independent experiments. (E). Summary of the phenotype detected in cdc6/c-myc double transgenic fish. EMT, epithelial to mesenchymal transition.

To provide more evidences to support the skin transformation phenotype, we performed plastic section through the tumor-like tissue in the pectoral fin of cdc6/c-myc transgenics. In the section crossing pectoral fin of WT with nlsEGFP-positive background, we found the EGFP-positive skin nucleus is flat and restrictedly located on the superficial layer (a in Fig. 6A). Intriguingly, some ectopic EGFP-positive skin cells were detected in the internal location of the pectoral fin of cdc6/c-myc transgenics (d in Fig. 6A, highlighted by yellow stars).

In addition, severe disorganization of the muscle and cartilage layers was also noticed in cdc6/c-myc transgenics (e in Fig. 6A). In many types of cancer, EMT transformation featured by down-regulation of epithelial markers and up-regulation of mesenchymal makers is frequently detectable and strongly associated with cancer malignancy [27]. The disorganization of skin cell distribution and the appearance of ectopic skin cells may suggest that the transformed skin cells are undergoing EMT. To clarify this speculation, we examined the mRNA expression profile of key genes related to EMT by real-time RT-PCR on embryos aged 8 dpf. Results showed that most epithelial markers, E-cadherin (*cdh1*) and type I keratin (*cki, cyt1, krt17, krt18*) and type II keratin (*krt5, krt8, krtt2c6,* and *krtt2c8*), were downregulated in cdc6/c-myc lines (Fig. 6B). In contrast, mesenchymal gene transcripts of *mmp9*, *snail1a*, *snail1b*, *snail2*, *twist1a,* and *twist1b* were significantly upregulated in cdc6/c-myc lines (Fig. 6C). We further confirmed this EMT phenomenon by immunofluorescent staining. Agreed with mRNA expression, the relative expression level of epithelial markers of cytokeratin 8 (CK8) and cytokeratin 13 (CK13) was robust expressed in WT while they were barely detected in cdc6/c-myc lines (a and b in Fig. 6D). Again, the mesenchymal maker MMP9 was highly expressed in cdc6/c-myc lines but not in WT (c and f in Fig. 6D). These results strongly suggest that co-overexpression of cdc6 and c-myc in zebrafish skin induces EMT, a hallmark of malignant invasion and metastasis in human cancer.

## DISCUSSION

In this work, we established a novel non-melanoma skin cancer model in zebrafish to study the oncogenic mechanisms of Cdc6 and c-Myc by co-overexpression of both in the outmost skin layer, rendering the practicability in a good cancer model and anti-cancer drug screening. Our studies suggest that co-overexpression of Cdc6 and c-Myc in zebrafish skin causes skin transformation through accelerated cell proliferation, apoptosis attenuation, genomic instability, and EMT (Fig. 6E).

In this study, we successfully created an early onset and a non-melanoma skin cancer model by specifically co-overexpressing two oncogenic genes that cause unbalance of cell cycle progression. Skin is the outmost tissue and provides a good model to evaluate the oncogenic transformation in living animal in a non-invasive manner. In addition, the outmost nature also makes it is easy to evaluate the biological effect of anti-cancer compound against cancer. In zebrafish, although many studies were attempting to induce skin cancer formation, no successful case was found in any skin cancer models except only in melanoma model [28]. Since we were failed to transform zebrafish skin cells by overexpression of single oncogene, we then tried to co-express two oncogenes to overcome the refractory threshold in zebrafish skin. Using co-overexpression of cdc6 and c-myc, we can induce tumor-like formation on the skin as early as from 8 dpf onwards even in the WT fish with a normal p53 expression background. This cdc6/c-myc transgenic line will be an excellent experimental animal model and an anti-cancer drug screening platform for identifying the potent c-Myc or Cdc6 inhibitor.

Our findings suggested that c-Myc only is able to induce skin hyperplasia but not sufficient to transform zebrafish skin. c-Myc is a transcription factor that directly modulates transcription of a large number of genes involved in cell cycle regulation, protein synthesis, metabolism, DDR, and apoptosis [29, 30]. The profound functions of c-Myc in tumorigenesis have been mainly attributed to its ability to coordinate transcription. In addition, deregulated expression of c-Myc leads to the generation of double strand breaks (DSBs), DDR and thus promotes genome instability [31]. Therefore, we suggest the reason of the failure of skin transformation induced by c-Myc overexpression only may be that cell transformation induced by oncogenic activation is restrained by cell cycle arrest, cellular senescence, and apoptosis, just like *ras* and *mos* [11, 12].

Next we found that co-overexpression of cdc6 and c-myc induces tumor-like transformation in zebrafish skin. The mechanisms could be increased cell proliferation, apoptosis attenuation, and the effect of genomic instability. Several explanations have been proposed to elucidate the phenomenon of genomic instability triggered by c-Myc overexpression, including inducing DNA replication stress [9, 32, 33], inhibiting DNA repair activities [34], and increased generation of reactive oxygen species (ROS) [25]. In addition, a recent study has identified a different mechanism for c-Myc in the direct stimulation of DNA replication, independent of its transcriptional targets. c-Myc is able to bind to components of the pre-RC, including Cdc6, and bind to known mammalian replication origins [9]. The genetic interaction between c-Myc and Cdc6 observed in the double transgenic zebrafish could be elucidated as the following. First, c-Myc and Cdc6 overexpression may enhance replication stress through both transcription-dependent and -independent function of c-Myc. Our results support the oncogenic potential of overexpressed c-Myc and Cdc6 from the earliest stages of skin transformation, which may be achieved by active replication stress. Initially, the stress activates the antitumor barriers of senescence or apoptosis (Myc or Cdc6 overexpression only), but the barriers eventually are bypassed by the continuous co-overexpression of c-Myc and Cdc6, which induces genomic instability favoring clonal expansion of cells with more aggressive properties. Second, Cdc6 overexpression may regulate the switch between the transcription-dependent and -independent function of c-Myc, as a study [13] showed that Cdc6 overexpression interferes with the interaction between c-Myc and Max, which inhibits E-box-dependent transcription activity of c-Myc. Third, Cdc6 overexpression may increase replication efficiency and gene expression of *c-myc* gene because a recent study showed that Cdc6 is increased its binding to the replication origins of *c-myc,* and the binding augments the origin activity [35].

Intriguingly, we observed that co-overexpression of cdc6 and c-myc induces EMT in zebrafish skin. Consistently, in cdc6/c-myc transgenic lines, the gene expression of epithelial markers, E-cadherin and cytokeratin, were downregulated; the gene expression of mesenchymal markers, MMP9, Snail, Twist1 were significantly upregulated. This result is supported by the finding that overexpressed Cdc6 binds directly to the E-boxes of the *Cdh1* promoter and represses its transcription [14].

## MATERIALS AND METHODS

### Fish lines and plasmid construction

The detailed procedure for fish lines and plasmid construction used in this study have been described in previous publication [36] and in Supplementary Data.

### Image Acquisition, Skin Cell Quantification and Statistics

Representative fluorescent images were acquired using an upright microscope (BX51, Olympus) equipped with a digital camera (DP72, Olympus) or a dissecting microscope (SMZ1500, Nikon) equipped with a cool CCD (Evolution VF). For quantifying the relative density of skin cells, the original images captured at the trunk position were processed using Photoshop CS3 software to select a region of interest (ROI) at 150 μm x 450 m dimensions. The total cell number in this ROI was calculated using ImageJ software and statistically compared using t-test or one-way ANOVA.

### Whole-Mount Immunostaining and Histology

Zebrafish embryos aged specific developmental stages were fixed in 4% paraformaldehyde/PBS for 12 h at 4°C. After extensive washing in PBST, embryos were transferred to 100% methanol at -20°C for 2h and subsequently subjected to rehydration with PBST. After blocking with 3% BSA/PBST at room temperature for 60 min, embryos were incubated at 4°C overnight with 1:200 diluted primary antibodies as follows: rabbit anti-pH3 (sc-8656-R, Santa Cruz), rabbit anti-human phosphorylated ribosomal Protein S6 (Ser235/236) (GTX113542, GeneTex), rabbit anti-human CK8 (GTX110311, Genetex), rabbit anti-human CK13 (GTX109883, Genetex), rabbit anti-human MMP9 (GTX100626, GeneTex), and rabbit anti-human CENPF (GTX100212, Genetex). After extensive washing in PBST for 10 min, embryos were incubated with 1:500 diluted Alexa Fluor 488 or 568-conjugated secondary antibodies (Invitrogen) for fluorescent detection of immunoreactive signals. For histology, some stained embryos were further infiltrated and embedded in Technovit 7100 resin (Heraeus Kulzer). Samples were sectioned at 3 μm intervals and counter-stain with either Hoechst 33342 (Invitrogen) or SYTOX orange (Invitrogen) to visualize the nuclear position.

### Real-time quantitative PCR

Ten zebrafish larvae aged at 8 dpf from wild-type or double transgenic lines were collected and homogenized in RNAzol RT (RN190, MRC, Inc) with Bullet Blender (Next Advance, Inc) tissue lyser to isolate total RNA according to the manufacturer's instructions. Total RNA concentration was determined by spectrophotometry, and the RNA quality was checked by electrophoresis in RNA denatured gels. For RT-PCR, 1 μg of total RNA was reverse-transcribed with RevertAid first cDNA synthesis kit (K1622, Fermentas) and then PCR was performed with SYBR green dye according to the manufacturer's instructions. The PCR condition: 95°C (15 s), 60°C (1 min), 72°C (1 min) for 40 cycles, and all reactions were started at 95°C for 5 min and terminated at 72°C for 5 min. The primer sequences used to perform RT-qPCR and the PCR amplicon size are listed in Table S1.

### BrdU Incorporation

Zebrafish embryos aged 7 dpf were systematically incubated in 10 mM BrdU (B5002, Sigma)/5% DMSO/PTU solution for 24 hr. After fixation, embryos aged 8 dpf were depurated with 2 N HCl for 60 min at room temperature and immersed in 0.1M borate buffer for 10 min to enhance the sensitivity for detection of the BrdU-positive cells in skin. Later, embryos were subjected to staining with mouse anti-BrdU antibody (1:100, G3G4, DSHB) and Alexa Fluor 488-conjugated goat anti-mouse secondary antibody (Invitrogen).

### TUNEL assay

For TUNEL assay, embryos aged 18 hour post-fertilization (hpf) were pre-treated with PTU and then processed to PFA fixation at 48 hpf. Following washing in PBST for 10 min they were then stored in 100% methanol at -20°C for over 2h. Embryos were incubated with 3% H_2_O_2_/MeOH for 10 min at room temperature. Subsequently, embryos were rinsed two times with PBS and incubated with labeling solution, 10μL of enzyme solution plus 90μL of label solution at 37°C for 2h following the kit instructions (Roche Applied Sciences). Embryos were then washed three times in PBS, for 5 min each time, at room temperature. Subsequently, embryos were incubated with 100μL of converter POD at 37°C for 30 min. Embryos were rinsed three times in PBS and incubated with DAB solution (SK-4100, Vector Laboratories) for 5 min.

### Image-based cytometry

To facilitate skin cell counting, WT or cdc6+c-myc double transgenics were crossed with Tg(krt4:mCherry)^cy9^. To enhance the detection sensitivity and reduce the total amount of embryos used to analysis the cell cycle and DNA content, we adapted an imaged-based cytometry approach by using NucleoCounter NC-3000 FlexiCyte machine (Chemometec, Denmark). Initially, ten embryos aged 8 dpf were slightly fixed in 4%PFA for 20 min at room temperature and then dissociated with 250 uL lysis buffer at 37°C for 10 min. The cell lysate was later incubated with 250 uL stabilization buffer to neutralize the pH value. After this cell dissociation process, the mCherry fluorescence is gone and we performed immunofluorescent staining with rabbit anti-mCherry primary antibody (1:200, GTX5788, Genetex) and goat anti-rabbit IgG-Alexa Flour 568-conjugated secondary antibody (1:500, Invitrogen) to recover the skin fluorescence. Finally, all dissociated cells were counter-stained with Hoechst 33342 (H1399, Invitrogen) at 5 ppm concentration at 37C for 1 hr to illuminate cell nucleus and then loaded onto chamber slide (NC-Slide A2) for taking images. The DNA content and cell cycle were analyzed Nucleoview NC-3000 software according to manufactory instruction (Chemometec, Denmark).

### Array CGH and Data Processing

Genomic DNA was isolated from WT and cdc6/c-myc transgenic embryos aged 8 dpf according to standard protocol [37]. Genomic DNA samples were then subjected to perform quality check by spectrophotometry (NanoDrop ND-1000, Thermo Fisher. Scientific, Inc.) and DNA electrophoresis. High quality genomic DNA which pass the quality check were labeled with Cy3 and Cy5 fluorescent dyes by using NimbleGen Dual-Color DNA Labeling Kit. Reference DNA was labeled with Cy5-random nonamers while test DNA was labeled with Cy3-random nonamers through klenow reaction. Hybridization assays were performed according to the manufacturers' instructions by using NimbleGen zebrafish CGH 385K Whole Genome-Tiling array. The zebrafish whole genome tiling array contains 65,536 probes which designed according to the Zv6 assembled genome. Hybridization reaction between probe and array was conducted with NimbleGen Hybridization Kit and NimbleGen Hybridization system to provide an active mixing action for 48hrs and constant incubation temperature at 42°C. The hybridization result was scanned with NimbleGen MS200 microarray scanner automatically taking optimized signal in 2μm pixel resolution and the relative fluorescent intensity was normalized, converted to log2 scale by NimbleScan software. The segmented CGH data were generated by CGH-segMNT analysis of NimbleScan software.

### Adenovirus construction and infection

Adenoviruses encoding GFP (Ad-Vec), human Cdc6 (Ad-Cdc6) and Cdt1 (Ad-Cdt1) were generated and purified by using the AdEasy system [38] and as described previously [39]. Large-scale purification of adenovirus from 293 cells was performed by CsCl density gradient centrifugation. The concentration of purified virus was measured OD_260_ using the equation 1OD_260_ ≈ 10^12^ pfu [40].

The detailed procedures for cell culture and microinjection and identification of transgenic Zebrafish used in this study are described in Supplementary Data.

## SUPPLEMENTARY MATERIALS AND METHODS TABLE AND FIGURES


