# Supplementary material for: Cdc6 cooperates with c-Myc to promote genome instability and epithelial to mesenchymal transition (EMT) in zebrafish
Source: Oncotarget. 2014 Jul 11;5(15):6300–11. doi: 10.18632/oncotarget.2204 (PMC4171631; doi:10.18632/oncotarget.2204)
Supplement: Supplementary file 1 [file oncotarget-05-6300-s001.pdf]

Cdc6 cooperates with c-Myc to promote genome instability and EMT in zebrafish

Supplementary Materials and Methods

Fish lines

Zebrafish were handling according to established protocol. The Tg(krt4:hcdc6)^{cy24}, Tg(krt4:Mmu.c-myc^{T58A})^{cy21}, and Tg(krt4:nlsEGFP)^{cy34} have been described in previous publication [1].

Plasmid Construction

Tol2 kit was used to rapidly assemble expression vectors by two-fragment gateway recombination cloning [2]. For cloning middle entry of pME-cdc6, the cdc6 open reading frame was amplified from human cDNA using forward primer (5'-GGGGACAAGTTTGTACAAAAAAGCAGGCTATGCCTCAAACCCGATCCCAGGC-3') and reverse primer (5'-GGGGACCACTTTGTACAAGAAAGCTGGGTTTAAGGCAATCCAGTAGCTAAG-3'). The *att*B1 and *att*B2 sites were added for forward and reverse primers, respectively, at the 5'end of primers and highlighted by underlines. The PCR products were cloned into pDONR221 (Invitrogen) to generate pME-cdc6. For cloning middle entry of pME-c-myc T58A, we performed BP reaction to move mouse c-myc T58A open reading frame from pMXs-c-Myc (Addgene 13372) into pDONR221 vectors. Finally, p5E-krt4, pME-cdc6 or pME-c-myc T58A and p3E-polyA were assembled together with pDestTol2CG2 by LR reaction to create two expression vectors of pDestTol2CG2-krt4-cdc6-pA and pDestTol2CG2-krt4-c-myc T58A-pA. For pTolDest-krt4-h2afv-mCherry-pA vector, p5E-krt4, pME-h2afv-mCherry were assembled with pTolDest by LR reaction.

Microinjection and Identification of Transgenic Zebrafish

Transposase RNA was synthesized *in vitro* using pCS-transposase plasmid (kindly provided by Dr. Koichi Kawakami) as a template. DNA was linearized with *Not*I at 37°C overnight and cleaned up using DNA Clean/Extraction Kit (GeneMark Inc., Taiwan). Capped mRNA was synthesized using mMESSAGE mMachine SP6 Kit (Ambion). For generation of transgenic zebrafish, expression constructs of pTolDest-krt4-

h2afv-mCherry-pA (50 ng/μL), pDestTol2CG2-krt4-cdc6-pA (50 ng/μL) and pDestTol2CG2-krt4-c-myc^{T58A}-pA (50 ng/μL) were mixed with *in vitro* transcribed transposases mRNA (50 ng/μL), and approximately 1-3 nL DNA solution was injected into the animal pole of one-cell stage embryos. The injected embryos were raised to adulthood and the putative founders were screened according to the green fluorescent signals in the heart of their F1 progenies. The transgenic fish line nomenclatures of Tg(krt4:h2afv-mCherry)^{cy9}, **Tg(krt4:Mmu.c-myc^{T58A})**^{cy21}, Tg(krt4:Hsa.cdc6)^{cy24} were approved by the Zebrafish Nomenclature Committee of ZFIN [3]. All experiments were approved by the animal use committee at Chung Yuan Christian University (approval ID: 9911).

Cell culture

U2OS cells were cultured in medium containing Dulbecco's modified Eagle's essential medium (DMEM), supplemented with 5% fetal bovine serum (FBS) and 5% super calf serum. The all medium contain penicillin 100 Units/mL, streptomycin 100 μg/ mL.

gene	Forward primer (5’ to 3’)	Reverse primer (5’ to 3’)	Amplicon (bp)
ccnd1	CTGATGGGTATGGGCTTTGG	TTTGGGCGTGCTGAGTGG	116
ccne	GGAATGGCTGGGAAAGGTTC	TTCTCGCTGCTTGGTGGTG	105
ccng1	AAAAAGCGCTGGTTGTTTGAAGC	CTAGGGTGGGAGTTAAGGGTTTC	149
cdk1	GTTGTACGCCTGCTAGATGTGC	ATTCGCCTGATGGGATGGA	109
cdk2	GATTACTCGGAGGGCACTGTTT	AGGTCCTGTCGAGCCCATTT	156
cdkn1a	TTGTCATGATTCTGTGGTCCCG	TTATTGACAGAAAACGACCTTCAG	169
cdh1	GTCGGATTATGCTATTGA	TAGTCTGTCTATCGTGAA	94
krt4	CTGTAACTGTCAACCAGAACC	CATCTTGTTCTGCTGTTCCA	161
krt5	TGCTTCCTTCATTGACAA	GGTAGTGGTCTGTTCTTG	91
krt8	ACAAGTCTAAGTATGAGGAGATG	CGGTTAAGGTCAGCGATT	181
krt17	GACTACAGCCACTATTATG	TTGTCAATCTGAAGGATG	95
krt18	AGGTCACAGAACACTACGA	GACAACAACACAACATAGAACAA	101
cki	AGACTGGAGATGGAGATT	TACTACTTTGCGTGTTGT	99
cyt1	TGGACAAGTCAATGTAGAG	TCATAGTGCTCACGGATA	81
krtt2c6	ATCAAGACTCAAGAGAAG	ATATCAATGTTGGACTGT	155
krtt2c8	AGCAGAGGTATGAAGATGA	CCAAGTATGTCGTGTCTAC	90
mmp9	TCGGCCTACCAAGCGACTT	TCATGTGAATCAATGGGCACTC	178
snai	CTTACTAGACTGTCAGGCT	CAATCCGTGCTTCTCGTA	93

1a	ATT		
snai1b	GATGCCACGCTCATTTCTT	GTCTGACTCTCCAGTTCACT	75
snai2	ACTTGTAACCATTGACTTCCAT	CTGTAACCACTGACTTCCATAG	100
snai3	GTGGTAACAGATAATTCCTTCA	GCTACAACAATAACAAGTATCAC	97
twist1a	TTCTGACAGCAACAACCA	TAACACTGATATGAGCCGATT	96
twist1b	CTTATGACGACGAATGTTG	CGAACTAGAGGCTTCAAT	161

Table S1.

Figure S1

A

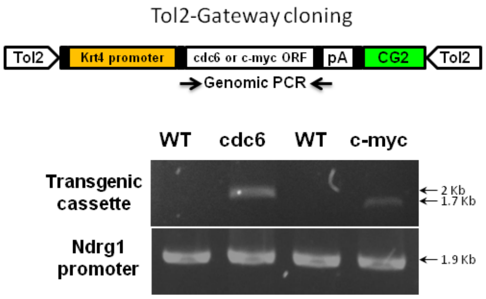

Ndrg1 promoter forward primer: GCGCATTGAATCTGTGGAGAG
Ndrg1 promoter primer: GCACAATCTTCCTCCAAGGACA

B

Transgene combination	Embryos with skin tumor	Tumor incidence rate (%)	Tumor initiation time (dpf)
WT	0/327	0	ND
cdc6	0/215	0	ND
c-myc	0/304	0	ND
cdc6/c-myc	34/228	15	8

Figure S1: Generation of transgenic fish and comparison of the tumor incidence rate in different transgene combination. (A) The schematic diagram in the upper panel shows the configuration of the plasmid used to generate transgenic fish. The parental vector of pDestTol2CG2, which contains *cmlc2*-EGFP-pA mini-gene, can facilitate transgenic fish screening by the presence of GFP signals in the heart. The lower panel illustrates the PCR results to confirm the transgenics used in this study carrying correct genetic background. Zebrafish Ndrg1 promoter as a positive control. (B) The summary of incidence rate of skin tumor detected in either single or double transgenics.

Figure S2

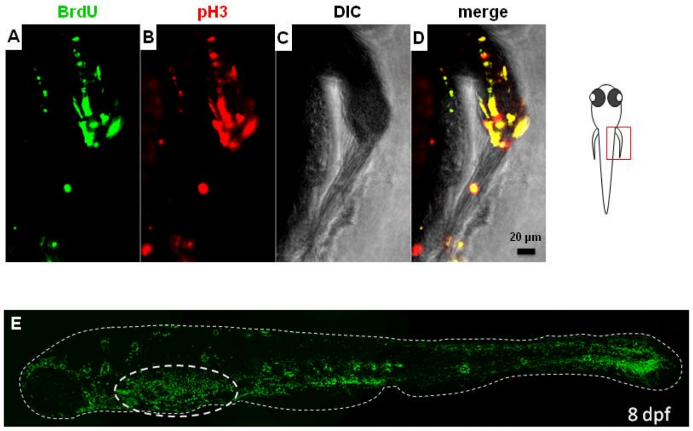

Figure S2: The comparison of cell proliferative ability between the pectoral fin and other body parts. The BrdU incorporation (A and E) and pHistone3 staining (B) experiments were conducted to compare the cell proliferation index between the pectoral fin and other parts in cdc6/c-myc double transgenic fish. The image highlighted by red box in the pectoral fin was magnified in A to D. (E) The comparison of BrdU+ cells between pectoral fin and other body parts in cdc6/c-myc zebrafish aged 8 dpf presented by the whole section of the body. The BrdU+ cells in the pectoral fin were highlighted by dotted circle.

Figure S3

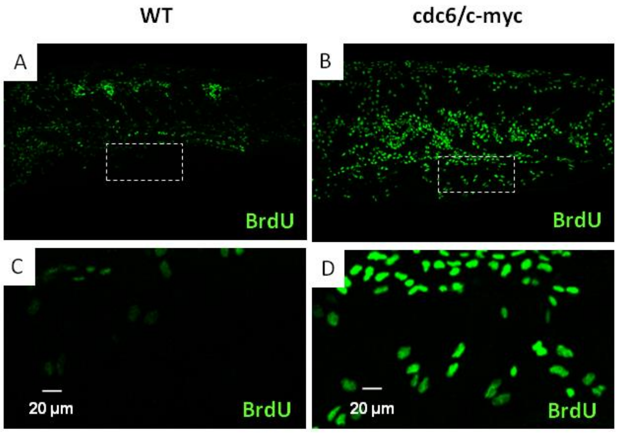

Figure S3: Comparison of cell proliferation in WT and cdc6/c-myc transgenic zebrafish. The BrdU incorporation experiment was conducted to compare the cell proliferation index between WT (A and C) and cdc6/c-myc transgenic fish (B and D). Embryos aged 7 dpf were incubated with 10 mM BrdU for 24 hr and later washed with fish water and finally fixed with PFA at 8 dpf and proceed for anti-BrdU antibody staining. The images highlighted by dotted lines in the ventral fin (A and B) were magnified in C and D, which show the cell patterns with positive BrdU-labeling. Scale bar =20 µm in C and D.

References

1. Chen, C. F., Chu, C. Y., Chen, T. H., Lee, S. J., Shen, C. N. & Hsiao, C. D. (2011) Establishment of a transgenic zebrafish line for superficial skin ablation and functional validation of apoptosis modulators in vivo, *PLoS One.* **6**, e20654.
2. Kwan, K. M., Fujimoto, E., Grabher, C., Mangum, B. D., Hardy, M. E., Campbell, D. S., Parant, J. M., Yost, H. J., Kanki, J. P. & Chien, C. B. (2007) The Tol2kit: a multisite gateway-based construction kit for Tol2 transposon transgenesis constructs, *Dev Dyn.* **236**, 3088-99.
3. Tanaka, S., Komeda, Y., Umemori, T., Kubota, Y., Takisawa, H. & Araki, H. (2013) Efficient initiation of DNA replication in eukaryotes requires Dpb11/TopBP1-GINS interaction, *Molecular and cellular biology.* **33**, 2614-22.
